# It's LiFe! Mobile and Web-Based Monitoring and Feedback Tool Embedded in Primary Care Increases Physical Activity: A Cluster Randomized Controlled Trial

**DOI:** 10.2196/jmir.4579

**Published:** 2015-07-24

**Authors:** Sanne van der Weegen, Renée Verwey, Marieke Spreeuwenberg, Huibert Tange, Trudy van der Weijden, Luc de Witte

**Affiliations:** ^1^ CAPHRI School for Public Health and Primary Care Department Health Services Research Maastricht University Maastricht Netherlands; ^2^ Research Centre Technology in Care Zuyd University of Applied Sciences Heerlen Netherlands; ^3^ CAPHRI School for Public Health and Primary Care Department Family Medicine Maastricht University Maastricht Netherlands

**Keywords:** motor activity, behavior change, self-management support, primary care nursing, remote sensing technology, COPD, type 2 diabetes

## Abstract

**Background:**

Physical inactivity is a major public health problem. The *It’s LiFe!* monitoring and feedback tool embedded in the Self-Management Support Program (SSP) is an attempt to stimulate physical activity in people with chronic obstructive pulmonary disease or type 2 diabetes treated in primary care.

**Objective:**

Our aim was to evaluate whether the SSP combined with the use of the monitoring and feedback tool leads to more physical activity compared to usual care and to evaluate the additional effect of using this tool on top of the SSP.

**Methods:**

This was a three-armed cluster randomised controlled trial. Twenty four family practices were randomly assigned to one of three groups in which participants received the tool + SSP (group 1), the SSP (group 2), or care as usual (group 3). The primary outcome measure was minutes of physical activity per day. The secondary outcomes were general and exercise self-efficacy and quality of life. Outcomes were measured at baseline after the intervention (4-6 months), and 3 months thereafter.

**Results:**

The group that received the entire intervention (tool + SSP) showed more physical activity directly after the intervention than Group 3 (mean difference 11.73, 95% CI 6.21-17.25; *P*<.001), and Group 2 (mean difference 7.86, 95% CI 2.18-13.54; *P*=.003). Three months after the intervention, this effect was still present and significant (compared to Group 3: mean difference 10.59, 95% CI 4.94-16.25; *P*<.001; compared to Group 2: mean difference 9.41, 95% CI 3.70-15.11; *P*<.001). There was no significant difference in effect between Groups 2 and 3 on both time points. There was no interaction effect for disease type.

**Conclusions:**

The combination of counseling with the tool proved an effective way to stimulate physical activity. Counseling without the tool was not effective. Future research about the cost-effectiveness and application under more tailored conditions and in other target groups is recommended.

**Trial Registration:**

ClinicalTrials.gov: NCT01867970, https://clinicaltrials.gov/ct2/show/NCT01867970 (archived by WebCite at http://www.webcitation.org/6a2qR5BSr).

## Introduction

Physical inactivity is a major public health problem [1,2] because it increases the risk of several diseases, such as coronary heart disease, type 2 diabetes, and several types of cancer. It also shortens life expectancy [1]. For people with a chronic disease, physical inactivity enhances the chance of complications and comorbidities [3]. Unfortunately, about one-third of adults worldwide do not reach public health guidelines for recommended levels of physical activity (PA) [4]. Therefore, the promotion of PA is a public health priority [5]. One of the approaches to increase PA is through primary health care [6]. Because practice nurses have frequent contact with people with chronic conditions to monitor treatment outcomes, it is recommended that they incorporate support to change physical inactivity behaviors [7,8]. However, providing only verbal advice has proven to be insufficient [9]. Despite the heterogeneity in results of physical activity intervention studies, the most effective approach is professional advice and guidance with continued support and combining a mix of behavior change strategies [10-12]. Effective behavior change strategies for the promotion of PA are self-monitoring, providing feedback for behavior, goal setting, providing tools to facilitate behavior, action planning, social support, barrier identification, and providing information on the consequences specific to the individual [10,11,13].

An example of a tool to facilitate behavior is the use of innovative technology such as mobile phones with built-in, or in combination with, pedometers or accelerometers. These technologies can facilitate self-monitoring, goal setting, and real-time feedback. Despite the fact that general mobile phone use is growing as well as mobile phone use in PA research [14], there is a lack of well-designed experimental studies with appropriate intervention periods and sample sizes [15] to explore whether these technologies add value on top of behavior change counseling by the practice nurse (PN). The *It’s LiFe!* intervention is a combination of behavior change strategies delivered by the PN in a self-management support program (SSP) that is partly integrated with usual care as well as the use of a monitoring and feedback tool for patients in daily life.

A cluster randomized controlled trial was conducted to evaluate the longitudinal effects of this multifaceted intervention on 40-70 year-old patients with chronic obstructive pulmonary disease (COPD) and diabetes type 2 (DM2) in primary care. Furthermore, the additional effect of using this tool on top of the SSP was evaluated. The main hypothesis was that after a 4-6 month intervention period, the complete intervention increases participants’ moderate to vigorous physical activity by at least 10 minutes per day compared to care as usual and that this increase maintains over 3 months.

## Methods

The study methods, intervention, and outcomes have been reported in detail previously [16]. See Multimedia Appendix 1 for the CONSORT-EHEALTH checklist [17].

### Study Design

A three-arm clustered randomized controlled trial among 24 general practices in the south of the Netherlands was conducted (NCT01867970). A cluster design was chosen to avoid contamination by unintended influence of the PN in the control group. After stratification based on the number of registered DM2 patients per practice, two blocks of 12 practices were randomly assigned to three groups using sealed envelopes. Practices allocated to Group 1 received the complete intervention (monitoring and feedback tool and SSP), practices in Group 2 received the SSP only, whereas practices in Group 3 received care as usual. Four strata were defined: small (<90 DM2 patients), medium (90-190), large (190-390), and extra-large (>390). There was no blinding for allocation of practices. The research team was blinded for allocation of participants during the analysis phase. Data were analyzed anonymously and coding was revealed after analyses.

### Participants: Practices and Patients

We invited 250 family practices in the South of Netherlands by invitation letter, telephone, or personal contact, until 24 practices agreed to participate. Eligibility for participants was determined as follows: between 40 and 70 years old with DM2 or COPD, and who did not, according to the PN, comply with the Dutch Norm for Healthy Exercise (having at least 30 minutes of moderate to vigorous physical activity on 5 or more days of the week) [18]. Additional inclusion criteria for the DM2 patients was a body mass index (BMI) >25, and for the COPD patients, a clinical diagnosis of COPD according to the GOLD-criteria stage 1-3, known to be stable in their respiratory function for at least 6 weeks, and on a stable drug regimen. Furthermore, participants needed to be able to access a computer with an Internet connection and master the Dutch language sufficiently.

Exclusion criteria were the presence of coexisting medical conditions with a low survival rate, severe psychiatric illness, or chronic disorders or diseases that seriously influence the ability to be physically active, and being treated primarily by a medical specialist or participating in another PA intervention. The PNs in each practice were asked to send 20-32 general invitation letters to patients who met the inclusion criteria. After randomization, the PN called the patients to give specific information about the allocated condition and ask if they wanted to participate. If the patient decided to participate, they received a specific information letter and an informed consent form. Each practice was instructed to include 5-7 patients with DM2 and 5-7 patients with COPD. This study was approved by the Medical Ethical Committee of the Maastricht University/Academic Hospital Maastricht in the Netherlands (12-3-071).

### Intervention

#### Overview

The complete *It’s LiFe!* intervention consisted of the self-management support program and a monitoring and feedback tool. Both elements were developed in a user-centered design process and tested on usability and feasibility [19-23]. Furthermore, 2 patient representatives from the Netherlands Asthma Foundation and the Dutch Diabetes Association participated in the research group to provide feedback on every aspect of the trial.

#### The Self-Management Support Program

**Figure 1:**
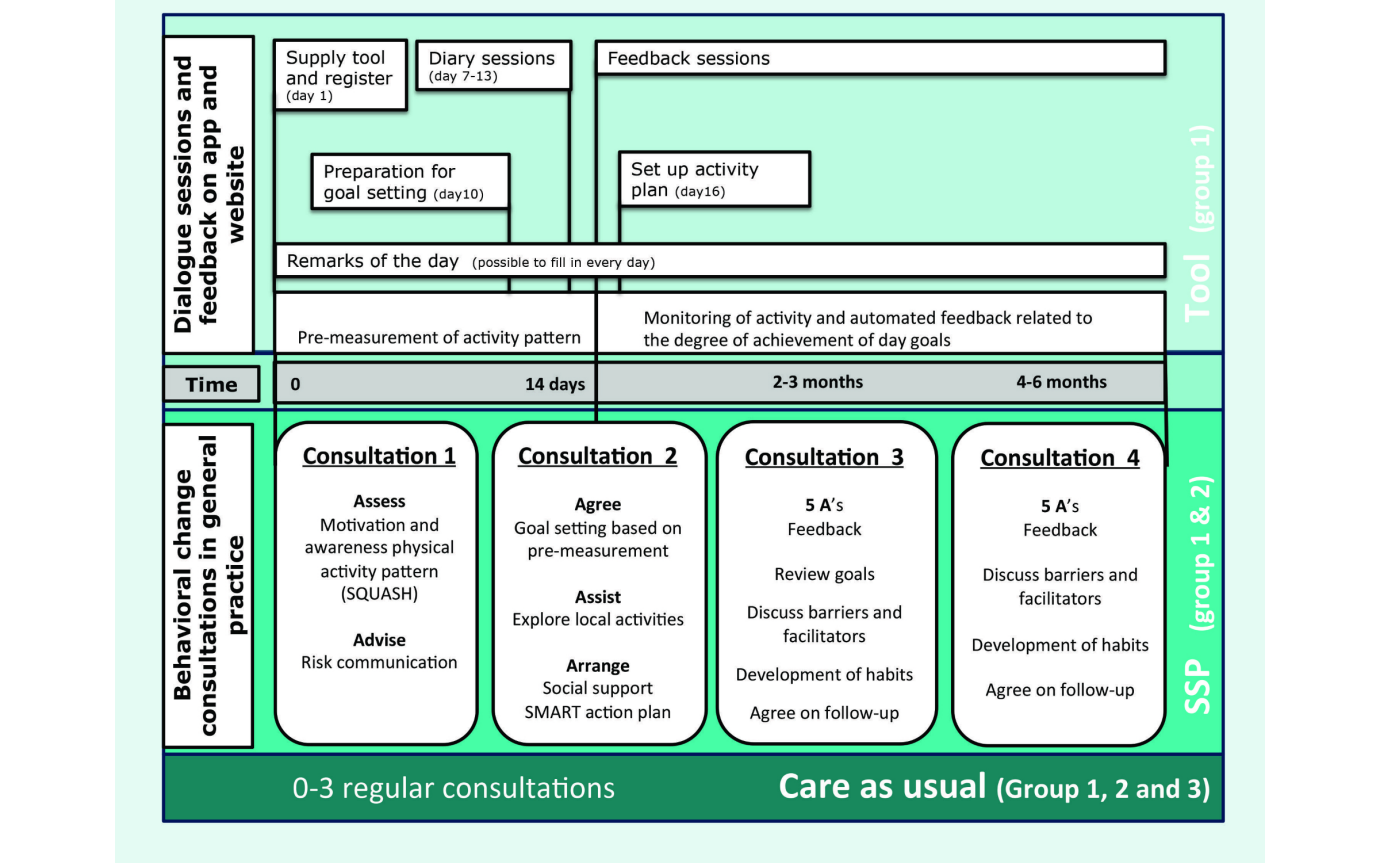
Course of the It's LiFe! interventions.

The program consisted of four individual consultations with the PN; in the first week, after 2 weeks, after 2-3 months, and after 4-6 months (Figure 1) [19]. First, the participants received an information booklet about the course of the intervention containing the Short Questionnaire to Assess Health-Enhancing PA (SQUASH) [24] and a list of locally organized PA activities.

In the first consultation, the PN raised awareness about the risks of physical inactivity, and the PA level of the patient was discussed using the previously completed SQUASH questionnaire. In addition, participants received a general and a disease-specific pamphlet about PA [25-27]. Between the first and the second consultation, a pre-measurement of the activity pattern was taken, and participants answered questions about barriers and facilitators for PA. In Group 1, PA was objectively measured by the tool, and all questions were answered via a dialogue session on the tool. Group 2 kept a PA diary on paper and answered questions about barriers and facilitators in the information booklet. During the second consultation, a personal goal was set in minutes of activity per day based on the pre-measurement, and the PN encouraged the participants to set up an activity plan to reach personal goals. Furthermore, the nurse informed the participants about locally organized PA options. In the third consultation, possibly by mail or telephone, activity results, barriers, facilitators, and the creation of new PA habits were discussed, and some participants reconsidered their activity goal. In the last consultation, activity results, barriers, facilitators, and PA habits were evaluated. Furthermore, how the PN and patient would continue the lifestyle coaching was discussed. The consultations were based on the “Five ‘A’s Cycle” counseling technique (assess-advise-agree-assist-arrange) [28,29].

#### The Tool

**Figure 2:**
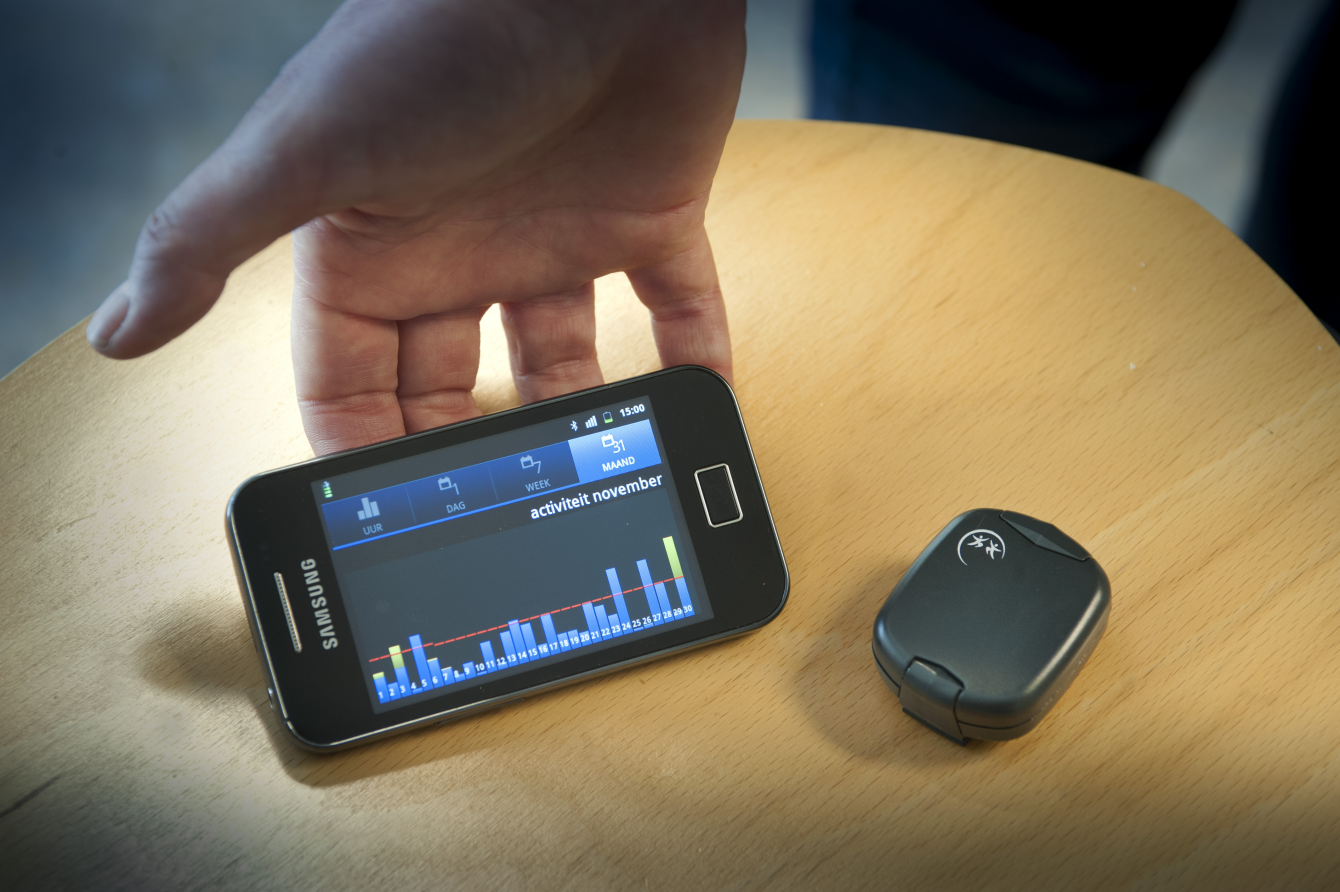
The It's LiFe! activity monitor and mobile phone app.

The tool consists of a three-dimensional (3D) activity monitor, a mobile phone app, and a Web app (Figure 2) [20]. Participants were asked to wear the activity monitor on a daily basis. They could see their real-time activity results and history in minutes of moderate to vigorous activity on the mobile phone and Web app, in relation to a personal goal. During the pre-measurement, participants participated in dialogue sessions (Figure 1). In the “diary sessions”, they were asked about enjoyment and exertion of performed activities. In the “preparation for goal setting”, they were asked about barriers and facilitators to exercise. Based on the activity results and the answers in the dialogue sessions, a personal activity goal was set in the second consultation of the SSP. Hereafter, automated feedback messages were sent related to the personal goal. Moreover, the participant was asked in a dialogue session to set up an activity plan to achieve the daily goal. During the entire intervention, activity results and answers to dialogue sessions were visible for the PN on a secured Web app [20,23]. The apps were not changed or updated during the trial (version 2.7). For technical questions and problems with the tool, the participants and PNs could contact a helpdesk during working hours to avoid contact between researchers and participants.

### Training of the Practice Nurses

For mastering the execution of the intervention, practice nurses in Groups 1 and 2 received an online Web lecture and consecutively a personal instruction session at their workplace. In addition, they received on paper, an explanation of the Five A’s model, the associated counseling techniques, and detailed instruction charts for each consultation. Nurses in Group 1 were able to try out the tool before the start of the consultations.

### Data Collection

All participants received a Personal Activity Monitor AM300 (Pam) [30-32] and questionnaires by regular mail, at baseline (t0), after the intervention at 4-6 months after baseline (t1), and 3 months after the end of the intervention, approximately 9 months after baseline (t2). The last measurement was initially set at 6 months after the intervention, but due to time and money constraints, this could not be realized. The Pam was blinded, which means that participants could not read the display with activity information to prevent any feedback and intervention effect of this measurement.

### Outcome Measures

The primary outcome measure was the average minutes per day of PA per patient, measured with the Pam [30-32]. The participants were asked to wear the Pam for 8 consecutive days clipped to their waistband on the hip and to record in a diary the time it was worn. A measurement was considered valid if the tool was worn on ≥5 days for ≥8 hours. Minutes per day were divided in three categories according to metabolic equivalent tasks (METS): light (1.8-2.99 METS), moderate (3-6 METS), and vigorous (>6 METS). The number of minutes of PA in the moderate and vigorous category (≥3 METS) was considered the primary outcome measure because moderate to vigorous activity is recommended by the World Health Organization [33]. Secondary outcome measures were general self-efficacy (general self-efficacy scale) [34], exercise self-efficacy (exercise self-efficacy scale) [35-37], and quality of life (RAND 36) [38,39].

### Statistical Analysis

The sample size calculation was based on the primary outcome measure (minutes of moderate to vigorous PA per day). Based on a power of 80%, an alpha of .05 (two-tailed testing), an expected difference between Groups 1 and 3 of 10 minutes of PA per day per participant, and an assumed intraclass correlation between the practices of 0.15, we required 72 participants over eight general practices in each group. A dropout rate of 10% was taken into account, which resulted in a desired number of 80 participants per group.

Intention to treat and per protocol analyses were performed. Participants of the intervention groups were included in the per protocol analysis if they received a minimum of three consultations (75%) spread over at least 3 months based on registration forms of the consultations obtained from the PNs. Participants from all groups were excluded from the per protocol analysis if they did not complete the second measurement (t1). Per protocol analysis were conducted to investigate whether results were different if only participants were included who adhered sufficiently to the interventions.

Normal distribution of the data was checked visually using normal q-q plots and histograms. Outliers were not removed. Continuous variables were presented as means, and standard deviation and categorical variables as numbers and percentages. Differences in baseline characteristics between groups at baseline were investigated with chi-square and analysis of variance (ANOVA). Variables that differed with a P value of .10 or smaller were considered as potential confounders in further analysis. For the RAND 36 outcomes, only the physical component and the mental component were used in further analysis, since the eight subscales strongly correlated. To adjust for the dependency of patients within time and practices (intraclass correlation [ICC]), we used restricted maximum likelihood (REML) multilevel analyses with random intercepts. The differences of the -2 log likelihood and degrees of freedom between models were examined to decide if a one-, two-, or three-hierarchical (time, participants, and general practices) model had to be applied (model selection was performed with a maximum likelihood [ML]). Separate models were set up for each outcome measure, adjusted with Bonferroni correction. The independent variables in each model were two dummy variables indicating the group, with the group of participants receiving care as usual as the reference category, and two dummy variables for time and their interaction effects. In addition, outcome estimates of the multilevel analyses were corrected for baseline and for potential confounders (differences between groups at baseline). Potential confounders were stepwise included in the model if the regression coefficients of time, group, and the interaction of group x time changed by ≥10% on average. To study whether the effects in COPD patients differed from the effects in participants with DM2, a subgroup analysis was done by including interaction effects. Missing values on items in questionnaires were handled according to the questionnaire’s analysis manual; missing data in follow-up were not imputed as multilevel analysis accounts for that [40]. All analyses were carried out with IBM Statistical Product and Service Solutions (SPSS) Statistics for Windows, version 22.0.

## Results

### Overview

**Figure 3:**
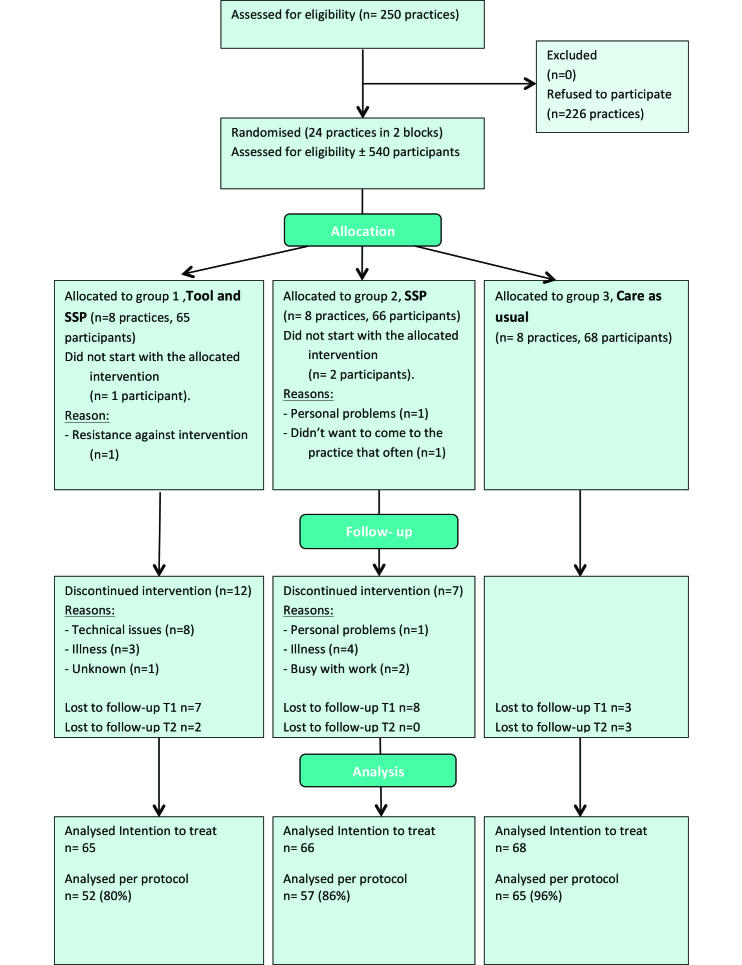
It's LiFe! CONSORT flow diagram.

In total, 24 general practices were randomly assigned to Group 1 (tool and SSP), Group 2 (SSP), or Group 3 (care as usual). In every group, we included one small practice, three medium, three large, and one extra-large practice. The individual practices included 3-14 participants with a median (interquartile range) of 9 participants (7-10 participants). As shown in Figure 3, PNs sent approximately 540 patients a general invitation letter and 199 patients (Group 1: 65 participants, Group 2: 66 participants, Group 3: 68 participants) agreed to participate and completed the baseline measurement. In June 2013, the first practices started with the intervention, and in April 2014 PNs in the last practices performed their last consultations. In Group 1, one participant did not start with the intervention because in his opinion, the intervention was not tailored to his age group, and 12 participants did not receive the minimal intervention as intended. In Group 2, 2 participants dropped out before the start of the intervention and 7 participants did not receive the minimal intervention as intended. In total, 23 participants were lost to follow-up. In the intention-to-treat analyses, data from all participants were taken into account (n=199) (Figure 3). Table 1 shows the baseline characteristics of participants in each group, and Table 2 shows the mean outcome values at baseline. Significant group differences, which were included as confounders in further analyses, were found for BMI, computer use, minutes of PA (≥3 METS), and quality of life (physical component scale).

**Table 1:** Baseline characteristics of participants.

Characteristics of participants		Group 1 (n=65), Tool & SSP	Group 2 (n=66), SSP	Group 3 (n=68), Care as usual
Female sex, n (%)		34 (52.3)	31 (47.0)	37 (54.4)
Age in years, mean (SD)		57.5 (7.0)	56.9 (8.3)	59.2 (7.5)
BMI^a^, mean (SD)		30.4 (5.7)	29.5 (5.9)	28.2 (4.3)
Origin non-Dutch, n (%)		5 (7.7)	4 (6.1)	3 (4.4)
Married or cohabiting partners, n (%)		48 (73.9)	46 (69.7)	55 (80.9)
**Education, n (%)**				
	Low	19 (29.2)	19 (28.8)	15 (22.1)
	Medium	35 (53.8)	40 (60.6)	43 (63.2)
	High	11 (16.9)	6 (9.1)	10 (14.7)
Employed, n (%)		31 (47.7)	31 (47.0)	31 (45.6)
COPD, n (%)		25 (38.5)	26 (39.4)	31 (45.6)
**Gold stadium, n (%)**				
	GOLD stadium 1	9 (36.0)	13 (50.0)	15 (48.4)
	GOLD stadium 2	15 (60.0)	12 (46.2)	16 (51.6)
	GOLD stadium 3	1 (4.0)	1 (3.8)	0 (0.0)
**Diabetes type 2, n (%)**		40 (61.5)	40 (60.6)	37 (54.4)
	Insulin use	3 (7.5)	6 (15.0)	8 (21.6)
**Comorbidities, n (%)**		51 (78.5)	46 (69.7)	43 (63.2)
	Asthma	6 (9.2)	8 (12.1)	4 (5.9)
	Cardiac/vascular	12 (18.5)	8 (12.1)	7 (10.3)
	Hypertension	22 (33.8)	29 (43.9)	20 (29.4)
	Arthritis	13 (20.0)	11 (16.7)	16 (23.5)
	Depression	3 (4.6)	5 (7.6)	5 (7.4)
	Also diabetes	2 (3.1)	1 (1.5)	1 (1.5)
	Also COPD	2 (3.1)	6 (9.1)	2 (2.9)
	Other	28 (43.1)	22 (33.3)	27 (39.7)
**Computer use** ^a^ **, n (%)**				
	Regularly	50 (76.9)	43 (65.2)	47 (69.1)
	Rarely	15 (23.1)	23 (34.8)	21 (30.9)
**Mobile phone use, n (%)**				
	Owns a smartphone	24 (36.9)	24 (36.3)	19 (28.0)
	Uses mobile phone frequently	20 (30.8)	20 (30.3)	15 (22.1)
	Uses mobile phone rarely	19 (29.2)	19 (28.8)	33 (48.5)
	Does not own a mobile phone	2 (3.1)	3 (4.5)	1 (1.5)

^a^ *P*≤.10, tested with chi square or ANOVA.

**Table 2:** Values at baseline.

			Group 1 (n=65), Tool & SSP	Group 2 (n=66), SSP	Group 3 (n=68), Care as usual
**Physical activity, mean (SD)**					
	Minutes per day in moderate and vigorous ≥3 METS^a^		39.3 (18.1)	47.5 (26.5)	44.1 (20.3)
	Wear time of the Pam in hours a day		14.3 (1.7)	14.5 (1.5)	14.3 (1.3)
**Self-efficacy, mean (SD)**					
	General self-efficacy scale		3.2 (0.5)	3.2 (0.5)	3.1 (0.5)
	**Exercise self-efficacy scale**		55.4 (17.0)	53.1 (21.3)	54.0 (19.2)
		Factor 1 Situational/interpersonal	51.2 (18.7)	45.9 (20.8)	48.3 (23.2)
		Factor 2 Competing demands	62.0 (18.5)	60.0 (21.6)	62.6 (20.2)
		Factor 3 Internal feelings	53.8 (18.8)	53.3 (22.2)	52.4 (21.1)
**Quality of life, mean (SD)**					
	Physical Component Score^a^		42.5 (11.1)	46.1 (9.8)	45.8 (9.4)
	Mental Component Score		48.2 (10.3)	48.6 (11.7)	50.1 (9.5)
		RAND36 physical functioning	68.7 (22.2)	74.6 (20.4)	74.7 (21.9)
		RAND36 role functioning physical^b^	55.8 (45.9)	72.2 (36.7)	70.8 (39.5)
		RAND36 role functioning emotional	72.8 (38.1)	77.4 (34.4)	78.4 (35.4)
		RAND36 social functioning	77.1 (22.8)	77.7 (23.8)	80.5 (20.8)
		RAND36 body pain	66.0 (24.8)	70.7 (25.1)	70.8 (23.1)
		RAND36 mental health	73.9 (15.1)	74.9 (19.7)	76.5 (14.9)
		RAND36 vitality^b^	55.2 (19.1)	62.5 (20.8)	64.3 (16.4)
		RAND36 general health	51.3 (19.6)	55.6 (20.6)	55.2 (16.2)

^a^ *P*≤.10, tested with ANOVA.

^b^ *P*≤.05, tested with ANOVA.

### Primary Outcome (Intention to Treat)

For the primary outcome, a two-level hierarchical model dealing with dependency of measurements in time within patients (but not family practices) was applied with a correction for baseline physical activity and wear time. ICC for repeated measures was .77, and ICC for participants in the same practice was .005. Directly after the intervention, participants in Group 1 who received the tool and the SSP showed 8 minutes more moderate and vigorous physical activity (≥3 METS) than participants in the SSP, and 12 minutes more PA than the care as usual group. This improvement difference was 9 minutes and 11 minutes, respectively, 3 months after the end of the intervention. No difference was observed between Group 2 (SSP) and Group 3 (care as usual). Results are shown in Table 3.

**Table 3:** Multilevel analyses for differences between the three groups for physical activity.

Follow-up		Unadjusted mean (SD)			Adjusted mean difference (95% CI); *P* value^a^			ICC^b^
		Tool & SSP	SSP	Care as usual	Tool & SSP: care as usual	SSP: care as usual	Tool & SSP - SSP	
PA moderate and vigorous (≥3METS)^a^	Baseline (t0)	39.29 (18.1)	47.47 (26.5)	44.13 (20.3)	-0.34 (-5.65 to 4.97); 1.000	0.15 (-5.13 to 5.44); 1.000	-0.50 (-5.83 to 4.84); 1.000	.77
	4-6 months (t1)	48.16 (23.8)	46.28 (30.8)	39.61 (19.5)	11.73 (6.21 to 17.25); .000^c^	3.87 (-1.60 to 9.24); .270	7.86 (2.18 to 13.54); .003^c^	
	9 months (t2)	48.82 (23.8)	45.34 (31.3)	42.40 (18.9)	10.59 (4.94 to 16.25); .000^c^	1.19 (-4.38 to 6.76); 1.000	9.41 (3.70 to 15.11); .000^c^	

^a^Adjusted for baseline physical activity and wear time.

^b^2-level random intercept (repeated measurements).

^c^ *P*<.01.

### Secondary Outcomes

For all secondary outcome measures, a two-level hierarchical model was applied. Table 4 shows that in general and exercise self-efficacy, no significant differences were observed. After 9 months, participants in Group 2 (SSP) did score significantly higher for the physical component of the quality of life scale than participants in Groups 1 (tool + SSP) and 3 (care as usual). At the end of the intervention (6 months), participants in both intervention groups did score significantly higher on the mental component scale compared to the care as usual group.

**Table 4:** Multilevel analyses for differences between the three groups for secondary outcome measures.

			Unadjusted mean (SD)			Adjusted mean difference (95% CI); *P* value^a^		
Follow-up			Tool +SSP	SSP	Care as usual	Tool +SSP – care as usual	SSP – care as usual	Tool +SSP- SSP
**Self-efficacy**								
	**General self-efficacy scale^b^**							
		Baseline (t0)	3.2 (0.5)	3.2 (0.5)	3.1 (0.5)	0.03 (-0.10 to 0.16); 1.000	0.03 (0.10 to 0.16); 1.000	-0.00 (-0.13 to 0.13); 1.000
		4-6 months (t1)	3.3 (0.4)	3.3 (0.5)	3.2 (0.4)	0.05 (-0.09 to 0.18); 1.000	0.02 (-0.11 to 0.15); 1.000	0.03 (-0.10 to 0.16); 1.000
		9 months (t2)	3.2 (0.5)	3.3 (0.5)	3.2 (0.4)	0.01 (-0.13 to 0.15); 1.000	0.00 (-0.13 to 0.13); 1.000	0.01 (-0.13 to 0.14); 1.000
	**Exercise self-efficacy scale^c^**							
		Baseline (t0)	55.4 (17.0)	53.1 (21.3)	54.0 (19.2)	1.10 (-5.04 to 10.38); 1.000	-0.68 (-8. 36 to 7.01); 1.000	2.67 (-5.04 to 10.38); 1.000
		4-6 months (t1)	59.7 (17.3)	59.7 (19.6)	54.5 (17.4)	4.86 (-3.12 to 12.83); .431	5.41 (-2.52 to 13.35); .304	-0.56 (-8.61 to 7.50); 1.000
		9 months (t2)	52.1 (16.1)	60.3 (19.1)	56.5 (19.2)	-0.03 (-8.01 to 7.94); 1.000	3.60 (-4.33 to 11.53); .828	-3.63 (-11.69 to 4.43); .838
**Quality of life**								
	**RAND physical component^d^**							
		Baseline (t0)	42.5 (11.1)	46.1 (9.8)	45.8 (9.4)	-0.31 (-2.48 to 1.86); 1.000	0.20 (-1.96 to 2.35); 1.000	-0.51 (-2.69 to 1.68); 1.000
		4-6 months (t1)	45.2 (9.5)	46.8 (10.0)	47.0 (10.0)	-0.07 (-2.32 to 2.19); 1.000	-0.08 (-2.33 to 2.17); 1.000	0.01 (-2.30 to 2.33); 1.000
		9 months (t2)	44.1 (9.5)	48.2 (8.6)	45.8 (9.5)	0.34 (-1.96 to 2.64); 1.000	2.99 (0.72 to 5.26); 0.005^e^	-2.65 (-4.99 to -0.32); 0.020^f^
	**RAND Mental component^d^**		
		Baseline (t0)	48.2 (10.3)	48.6 (11.7)	50.1 (9.5)	-0.30 (-3.27 to 2.68); 1.000	-0.39 (-3.34 to 2.56); 1.000	0.09 (-2.90 to 3.09); 1.000
		4-6 months (t1)	48.8 (10.6)	51.6 (11.3)	47.7 (9.8)	3.23 (0.14 to 6.32); 0.04^f^	4.39 (1.32 to 7.47); 0.002^e^	-1.16 (-4.33 to 2.01); 1.000
		9 months (t2)	48.3 (11.7)	50.1 (10.9)	50.3 (8.3)	0.21 (-2.94 to 3.36); 1.000	0.23 (-2.88 to 3.34); 1.000	-0.02 (-3.22 to 3.17); 1.000

^a^Linear mixed model 2-level random intercept (repeated measurements).

^b^Adjusted for baseline general self-efficacy scale, computer use, and baseline physical activity moderate + vigorous.

^c^Adjusted for baseline exercise self-efficacy scale.

^d^Adjusted for baseline RAND physical component and baseline RAND mental component.

^e^ *P*<.01.

^f^ *P*<.05.

### Per Protocol Analyses

The results from 174 participants (Figure 3) were analyzed for the per protocol analysis. All per protocol analysis confirmed the intention to treat analysis.

### Subgroup Analyses

No differences were observed in outcomes for people with COPD or type 2 diabetes (results not presented).

## Discussion

### Principal Findings

The complete *It’s LiFe!* intervention led to significant improvement of moderate to vigorous physical activity among patients with COPD or type 2 diabetes between 40 and 70 years old in primary care, compared to usual care. Right after the intervention period, the entire intervention added 12 minutes per day of moderate to vigorous physical activity compared to care as usual. Three months after the intervention period, this progress was still significant (11 minutes). This study also proved that use of the tool on top of the SSP is more effective than SSP only. The added value of the tool was an additional 8 minutes of moderate to vigorous physical activity per day. The SSP alone had no significant effect on physical activity compared to care as usual. For the secondary outcome measures, the intervention effect was not evident. It did not result in higher self-efficacy levels. Only the scores on the mental component scale of quality of life showed higher levels directly after both interventions, compared to care as usual, but this difference was not maintained after 9 months. At 9 months follow-up, participants in the SSP group scored significantly higher on the physical component of the quality of life scale compared to the other groups.

### Comparison With Prior Work

From the result that the tool embedded in the SSP is effective in contrast to the SSP alone, we can conclude that the automated self-monitoring and feedback component and/or the fact that the PN could see the objective measured PA results, was the most powerful element of the combined intervention. This is in line with the conclusion of a meta-analysis, that PA intervention studies for chronically ill patients incorporating self-monitoring showed a greater effect than studies without self-monitoring [41]. In the SSP, participants only monitored their behavior during the first 2 weeks by using an activity diary. The fact that PA was measured objectively in Group 1 may also have reinforced the goal setting component. Goal setting is more effective if goals are set with a specific outcome, proximal in terms of attainment, and realistic for the individual [13]. This is easier to achieve if objective PA results are available for the patient and the PN, and goals can be adapted during the intervention period based on the obtained results. The individual effect of the tool without the guidance by the PN cannot be extracted from this research, although we do expect that guidance by the nurse is an essential element of the intervention for first raising awareness, risk communication, social support, perseverance with the intervention, and adoption and persistent use of the tool. From the pilot study, we learned that participants felt a desire to succeed due to the commitment they made with the PN and the effort she put into them [22]. Other research also showed the importance of professional advice and guidance with continued support for the improvement of physical activity levels [12].

Other studies demonstrated that a reduction in the number of contacts diminished the behavior change that had been already achieved, especially when the intervention ends [13,42,43]. In this study, 3 months after the intervention period, Group 1 was still significantly more active than the care as usual and the SSP group.

Although exercise self-efficacy is positively correlated with physical activity levels [35], no significant differences were found on this scale between the groups nor on general self-efficacy. This is in line with the findings from the *It’s LiFe!* pilot study [22]. Surprisingly, no effects were found on the physical component of the quality of life scale directly after the intervention, but it did improve in the SSP group 3 months after the intervention. We have no explanation for this observation. Awareness that physical activity is being monitored might influence habitual behavior [44]. For the intervention, this was a desirable effect of the *It’s LiFe!* tool. However, it was an undesired effect of the use of the Pam. In this view, the proven effectiveness of the total intervention on the primary outcome—moderate to vigorous physical activity—is even more distinct considering the fact that those participating in research often show social desirable behavior while wearing an accelerometer for a short period of time [45]. Participants in Group 1, however, became used to being observed with an accelerometer for 4-6 months, which could have led to less socially desirable behaviors during the research measurement periods, compared to the other groups.

### Strengths and Limitations

To the best of our knowledge, this is the first randomized controlled trial that tests the added value of a monitoring and feedback tool in addition to counseling by the PN. An important strength of this study is the objective measurement of the primary outcome measure—physical activity—by an activity monitor instead of a subjective questionnaire. Other strengths are randomization at the practice level to minimize contamination, delay of randomization until after inclusion of the participants, the minimization of Hawthorne effects by avoiding contact between the researchers and participants, and simultaneous with the effect study, a process evaluation was conducted. The latter revealed that despite technical difficulties, the intervention was carried out as intended by the PNs. Another strength of this study is the pragmatic approach. Since the interventions were adapted and embedded in care as usual, it is more likely that the effects will be sustained in the daily primary care setting [46].

Limitations of this study were that the mean baseline physical activity was above the recommended level of 30 minutes of moderate to vigorous activity a day, only 10% of the approached family practices agreed to participate in the study, and only 37% of the approached patients agreed to participate in the intervention. These factors may have induced a selection bias, which makes the results less generalizable. However, a common reason for family practices to refuse participation was the required time investment for the practice nurse. Part of the time investment was for research purposes, which will be eliminated if embedded in daily practice. The low reach among patients may be explained by the fact that in this study patients with low physical activity levels who were not aware of the problem of their inactivity (according to the transtheoretical model of behavior change [47], the precontemplation phase of change) were not included, because the decision to participate had to be made before the consultation with the PN to create awareness could have taken place. In daily practice, the PN starts with raising awareness in regular consultations, which may result in a shift to the contemplation or preparation phase of change, and after this, patients will be asked if they are willing to work on their lifestyle with the help of the *It’s LiFe!* intervention. Another limitation of this study was that cycling, swimming, strength training, and all upper body movements were not taken into account in the primary outcome measure because these could not be captured with the Pam. Furthermore, the follow-up was relatively short—3 months after the intervention period. Ideally, a 12-month follow-up is recommended [48]. Due to time constraints, this was not possible. Clinical outcomes were not measured to avoid the Hawthorne effect in the care as usual group.

### Implications for Practice and Future Research

Results of this study revealed the powerful addition of continuous support by the use of a monitoring and feedback tool in addition to behavior change counseling. Because of this added value, it seems worthwhile to implement the intervention on a larger scale. However, cost-effectiveness should be investigated. To encourage general practices to adopt this intervention, health insurance companies should stimulate self-management support regarding physical activity with financial reimbursements for general practices. The fact that the availability and use of smartphones and wearables to measure physical activity is growing [49] is promising for the adoption of the intervention. In daily practice, the intervention can be easily tailored to the individual needs of the patient—for example, more time for raising awareness or referral to an exercise program with a physiotherapist if exercise self-efficacy or capacity is considered too low. In addition, the intervention can be more extensive or recurrent in care as usual with more emphasis on habit formation, instead of a determined period of 4-6 months. The application of this intervention to other target groups should be investigated just as the execution by other care providers as physiotherapists and dieticians.

### Conclusions

The monitoring and feedback tool, if embedded into a counseling protocol, was an effective instrument to improve physical activity of patients with COPD or type 2 diabetes between 40 and 70 years old. This improvement was sustained for 3 months. Counseling without the tool was not effective. The use of technology added to counseling is promising for physical activity behavior change. Future research about the cost-effectiveness and application under more tailored conditions and in other target groups is recommended.

## Appendix

Multimedia Appendix 1 CONSORT-EHEALTH checklist V1.6.1 [17].

## Abbreviations

BMI: body mass index
COPD: chronic obstructive pulmonary disease
DM2: type 2 diabetes
GP: general practitioner
*It’s LiFe!*: Interactive Tool for Self-management through Lifestyle Feedback
METS: metabolic equivalents
ML: maximum likelihood
PA: physical activity
Pam: Physical Activity Monitor AM300
PN: practice nurse
REML: restricted maximum likelihood
SSP: self-management support program
